# Cognitive and Language Deficits in Multiple Sclerosis: Comparison of Relapsing Remitting and Secondary Progressive Subtypes

**DOI:** 10.2174/1874205X01812010019

**Published:** 2018-03-12

**Authors:** Katerina Ntoskou, Lambros Messinis, Grigorios Nasios, Maria Martzoukou, Giorgos Makris, Elias Panagiotopoulos, Panagiotis Papathanasopoulos

**Affiliations:** 1 Rehabilitation unit for Patients with Spinal Cord Injury, “Demetrios and Vera Sfikas”, Department of Medicine, University of Patras, 26504 Patras, Greece; 2 Neuropsychology Section, Department of Neurology, University of Patras Medical School, 26504 Patras, Greece; 3 Higher Educational Institute of Epirus, Department of Speech and Language Therapy, Ioannina, Greece; 4 Higher Educational Institute of Peloponnese, Department of Speech and Language Therapy, Patras, Greece; 5 University of Patras Medical School, Patras, Greece

**Keywords:** Multiple sclerosis, Impairment, Cognition, Language, Relapsing remitting MS, Secondary progressive MS

## Abstract

**Objective:**

The objective of this study was to investigate the pattern and severity of cognitive and language impairment in Greek patients with Relapsing-remitting (RRMS) and Secondary Progressive Multiple Sclerosis (SPMS), relative to control participants.

**Method:**

A prospective study was conducted in 27 patients with multiple sclerosis (PwMS), (*N*= 15) with RRMS, (*N*= 12) with SPMS, and (*N*= 12) healthy controls. All participants were assessed with a flexible comprehensive neuropsychological – language battery of tests that have been standardized in Greece and validated in Greek MS patients. They were also assessed on measures of disability (Expanded Disability Status Scale; EDSS), fatigue (Fatigue Severity Scale; FSS) and depression (Beck Depression Inventory - fast screen; BDI-FS).

**Results:**

Our results revealed that groups were well matched on baseline demographic and clinical characteristics. The two clinical groups (RRMS; SPMS) did not differ on overall global cognitive impairment but differed in the initial encoding of verbal material, mental processing speed, response inhibition and set-shifting. RRMS patients differed from controls in the initial encoding of verbal material, learning curve, delayed recall of verbal information, processing speed, and response inhibition. SPMS patients differed in all utilized measures compared to controls. Moreover, we noted increased impairment frequency on individualized measures in the progressive SPMS group.

**Conclusion:**

We conclude that MS patients, irrespective of clinical subtype, have cognitive deficits compared to healthy participants, which become increasingly worse when they convert from RRMS to SPMS.On the contrary,the pattern of impairment remains relatively stable.

## 1 INTRODUCTION

Multiple Sclerosis (MS) is considered as being the primary demyelinating disease of the Central Nervous System (CNS). The disease usually occurs during the most productive years of human life, namely in the third and fourth decade, bringing with it significant socio-economic and interpersonal effects. Although the mean age of onset of symptoms is around 30, initial symptoms occur before the age of 16 in almost 5% of patients and after the age of 50 in approximately 10% [1]. As for the demographic factors, MS is two to three times more common in women than in men [2].Latitude seems to have a significant impact on a disease outbreak in that there is an increased prevalence of the disease in the temperate zones of the Earth, while prevalence declines closer to the tropical zone [3, 4]. As for the prevalence rate of MS cases in Greece, it has increased rapidly˙ from 29.5 per 100,000 population in 1990 (Northern Greece [5]) to 38.9 per 100,000 in 1999 (Northern Greece [6]) and to 119.61 per 100,000 population at the end of 2006 (South-western Greece - [3]). Thus, the average incidence of MS in Greece has increased nearly up to five-fold over the past 23 years.

Conventionally, MS is divided into three clinical types: Relapsing Remitting (RRMS), Secondary Progressive (SPMS) and Primary Progressive (PPMS) [7]. Symptoms of the disease vary greatly depending on the area of the CNS affected. Cognitive deficits have been reported in all stages and clinical types of MS [7, 8]. The typical pattern of cognitive impairment is the reduced speed of information processing, decreased phonological and semantic speech fluency output, deficits in verbal and visual episodic memory, attention and executive dysfunctions [9]. On the other hand, language function seems to remain relatively intact [10].

Several neuropsychological studies have compared the cognitive functions of patients between the three clinical types. More specifically, Denney *et al*. [11] found poorer cognitive functions in the progressive types of MS compared to the RRMS type and more severe cognitive deficits in SPMS, compared to the PPMS. Moreover, in a recent study conducted by Katsari *et al*. [12], results revealed deficits in episodic memory and executive functions only in patients with SPMS. On the other hand, Rosti-Otajarvi *et al*. [13] found a more severe cognitive decline in patients with PPMS, compared with patients diagnosed with the other two types. Similarly, Potagas *et al*. [8], in a sample of Greek-speaking patients with MS, found that patients with all three types of MS present cognitive deficits compared to a group of demographically matched healthy participants, whereas the performance of patients with PPMS was poorer compared with the performance of patients with the other two types.

As for communication disorders in MS, they almost exclusively present as speech-perceived disorders and dysarthria [14], whereas language impairment appears less frequently, although, in some cases, even aphasic deficits have been reported [15, 16]. Language impairments usually involve poor word recall and verbal fluency (phonological and semantic) [17]. Although at the clinical level, MS patients' performance in phonological fluency tests seems to be more disturbed than their performance in semantic fluency tests, Henry and Beatty [18], in a quantitative review of 35 studies with MS patients who had been examined in both tests, did not find significant differences in patients' performance between the two tests. The authors also reported that patients with SPMS had more severe verbal fluency deficits, compared with RRMS patients [18].

Since verbal fluency tests are significantly influenced by executive functions (*i.e*.cognitive strategies utilized to maximize word generation), deficits observed are probably due to a dysfunctional executive syndrome [17]. Moreover, since these tests are influenced by mental processing speed, and verbal storage skills, the observed impairments are not purely linguistic. This claim is in line with Messinis *et al*.'s [19] study, which investigated the differences in verbal fluency between patients with RRMS and SPMS, using the Greek phonological verbal fluency test. Authors confirmed the significant contribution of the executive strategy known as “switching”, which was used to maximize word production, in the differences attested in the performance of the two groups.

Although there has been an increase in the number of studies which have explored linguistic function over the past decade, the number of studies which compare performance among patients with the three types of MS remains limited and the results are contradictory ([20]). In a recent study [21], Greek-speaking patients with RRMS and healthy controls with similar demographic characteristics to those of the patients, were compared in the naming of verbs and nouns, to explore whether the capabilty of naming verbs was influenced by the semantic and phonological subtype of the verb. The results showed that MS patients experienced significantly more difficulty in recalling verbs compared with the control group. Further analysis revealed a statistically significant difference between the production of instrumental and non-instrumental verbs, with instrumental verbs being recalled with greater difficulty.

Therefore, it seems that the findings on cognitive and language dysfunctions among the three clinical types of MS remain contradictory, whereas there is only one study which explores this issue in native speakers of Greek [21]. The present study aims to add further data by examining cognitive and linguistic functions between patients with RRMS and SPMS, using a flexible neuropsychological - languagebattery of tests.

In this respect we hypothesized that: (a) participants with SPMS are anticipated to present with a qualitatively different pattern and quantitatively more severe cognitive and language impairments compared to patients with RRMS and healthy participants (b) mild to moderate correlations were expected to be found between clinical and demographic variables and performance in the neuropsychological - language tests.

## 2 METHODS

### 2.1 Participants

Twenty-seven patients with MS, 15 with RRMS and 12 with SPMS, and 12 healthy participants (Control Group - CG) took part in the present study. The diagnosis of MS was made in accordance with McDonald's revised criteria (for a detailed description of the criteria see [22]). Participants' demographic and clinical characteristics were recorded or evaluated (gender, age, years of education, medication and premorbid intelligence) and their clinical characteristics (severity and duration of the disease, fatigue and depression levels).

Patient’s inclusion criteria were: 1) to have been diagnosed with MS by an experienced neurologist, and 2) to have been clinically evaluated, based on the Expanded Disability Status Scale (EDSS), with a disability level ranging from 0 to 5. The criteria for participation for both patients and healthy participants were: 1) to have no history of other neurological disorders (*e.g*. stroke, epilepsy, encephalitis or severe traumatic brain injury), 2) to have no dementia and their score in the Mini Mental State Examination (MMSE) to be greater than or equal to 24 [23] 3) to have no history of major psychiatric disorders or psychotic symptoms (hallucinations, delusions) 4) to be native speakers of Greek 5) to be adults up to the age of 55 6) to have no presence of relapses or any change in EDSS score over the last six months before their participation in the study, 7) to have normal or corrected vision and hearing, and 8) not alcohol abuse or abuse of illegal drugs or steroids. Written consent was obtained from all participants of the present study after having been informed of the nature of the study they would take part in.

### 2.2 Procedure

After the approval of the present research protocol by the Ethics Committee of the University Hospital of Patras, evaluation of participants was conducted based on a flexible neuropsychological – linguistic test battery. The clinical evaluation of the patients was performed during a period of disease inactivity. Each patient underwent a comprehensive neurological, neurobehavioral, neuropsychological and language assessment, conducted by an interdisciplinary team (neurologists, clinical neuropsychologists, speech therapists), at the Neuropsychology unit, Department of Neurology, University Hospital of Patras.

### 2.3 Neuropsychological – Language Assessment

Neuropsychological and language assessment was performed by using the following tests:

1. For the assessment of the general mental state, the Mini Mental State Examination (MMSE) was administrated. MMSE is a test of general mental state assessment, which evaluates memory, attention, orientation, visual-spatial and language skills. MMSE is useful only as a cognitive screening [24]. A validation study of the MMSE in the Greek population was conducted by Fountoulakis *et al*. [23].
2. For the evaluation of verbal learning and memory, the Rey Auditory Verbal Learning Test (RAVLT) was used. The RAVLT evaluates the person's ability to encode, consolidate, store and retrieve verbal information. The administration was conducted in the following form: A 15-word list (list A) was orally presented and repeated five times. After the last of these learning tests, a new list of 15 different words (List B or Distracter List) was presented only once. First, participants' ability to recall the second list was evaluated, and then participants were instructed to recall as many words as they could from the first list. After 25 minutes, a free recall test was performed and then a memory recognition test using a wordlist containing target words, namely previously presented items, and new words that acted as distracters [25]. The dependent variable in this study was the average number of total words recalled in the five tests.
3. For the assessment of verbal expression / fluency, the Greek verbal fluency task was used. The Greek verbal fluency task assesses the effectiveness of thought and it is found to be sensitive to dysfunction of the left frontal cortex. In this test, participants are asked to orally produce as many different words as possible within 60 seconds belonging to three predetermined categories (semantic fluency) or beginning with three designated letters (phonological fluency) (see also COWAT in Minimal Assessment of Cognitive Functioning in MS - MACFIMS) [7, 26, 27, 28].
4. The Symbol Digit Modalities Test (SDMT) was used to measure cognitive processing speed and active memory. The SDMT is a substitution task in which participants by using a reference key, have 90 seconds to pair specific numbers with given geometric figures. It is one of the most sensitive tests for detecting cognitive deficits in MS and it is part of the MACFIMS battery. The detailed description of the original SDMT is available in Smith *et al*.'s [29] Clinical Manual, whereas the corresponding Greek norms can be found in Argirokastritou *et al*.'s [30] study.
5. To assess attention, visual-motor speed and mental processing speed, as well as set-shifting ability, the Trail Making Test (TMT), part A and part B were used respectively. In Part A, examinees are instructed to connect, by drawing lines on a sheet of paper, a set of 25 circled numbers in a numerical sequence as fast as possible. In part B, participants have to connect circled numbers (from 1 to 13) and letters (from A to M) in an alternating numeric and alphabetic sequence, as quickly as possible (*e.g*. 1-A, 2-B, *etc*.) (See also [31], for Greek norms see [32]).
6. For the assessment of response inhibition, the Colour - Word task of the Stroop Neuropsychological Screening Test (SNST) test was used. In the Colour-Word task participants are presented with printed coloured names which are not printed with a matching colour (*e.g*. RED is printed in blue ink) and they are instructed to ignore the verbal content of the word and to name aloud, as rapidly as possible, the colour of the ink in which the words are printed. The difficulty lies in suppressing an ordinary answer (*i.e*. reading the words), for the sake of a less common answer (*i.e*. the naming of the colour of the ink in which each word is printed). The score in this test depends on the number of correct answers in a period of 120 seconds. Poor performance reveals that participants have a selective attention disorder and thus they are unable to ignore misleading stimuli (interference). In another sense, the test evaluates an individual's ability to inhibit an automated response and to establish and maintain a new, unusual pattern of response. The analytical description of the test is available in Trenerry's [33] clinical manual, whereas the corresponding Greek norms are available in the studies conducted by Zalonis *et al*. [34] and Messinis *et al*. [35].
7. To evaluate the severity of depressive symptoms, the Beck Depression Inventory - Fast Screen (BDI-FS [36], was administered. This short version of the Depression Scale is suitable for assessing the presence and severity of depression in patients since it isolates the cognitive from the physical symptoms of depression. The latter may be overlapped with organic symptoms due to neurological disease (*e.g*., insomnia) [36]. The validity of the scale has been confirmed in patients with MS [37]. The BDI-FS has been translated and adapted to Greek at the Neuropsychology Laboratory of the Psychiatric Clinic, University Hospital of Patras (see [38])
8. The Fatigue Severity Scale (FSS [39], for Greek participants see [40]) was used to evaluate fatigue. This scale consists of 9 questions and estimates the level of fatigue experienced by patients over the last two weeks before their evaluation.

### 2.4 Statistical Analyses

All data were collected and processed by using SPSS (version 23). The Shapiro-WilkTest was used to evaluate the normality of data distribution. For normally distributed data, parametric criteria/tests were used, whereas for non-normally distributed data, non-parametric criteria were used. The comparison of demographic characteristics between the three groups was performed using a one-way ANOVA, for the variables age and years of education. *Pearson's chi-square* was used for the variable gender and the *independent sample t-test* for the variables WASI vocabulary score, disease duration, FSS (level of fatigue) and severity of depression (BDI-FS). The Mann–Whitney non-parametric U test for rank data was used to compare EDSS (disease severity). To evaluate whether there was a significant difference between the performance of the three groups in each of the neuropsychological - language tests, we used the one-way Analysis of Variance which compares the means of three or more independent groups. Post-hoc tests with Bonferroni correction were used for pairwise multiple comparisons between the groups. Effect sizes were calculated with Cohen’s *d* and Hedges *g* using the formula Cohen's *d* or Hedges *g* = (*M*_2_ - *M*_1_) ⁄ *SD*_pooled;_ *SD*_pooled_ = √ (*SD*_1_^2^ + *SD*_2_^2^) ⁄ 2). Both Cohen's *d* and Hedges' *g* pool variances on the assumption of equal population variances, but g pools using n - 1 for each sample instead of n, which provides a better estimate, especially the smaller the sample sizes. Hedges's *g *is a somewhat more accurate version of Cohen's *d* (with pooled SD) in that we add a correction factor for small samples. For very small sample sizes (<20) it is usually preferable to choose Hedges’ *g* over Cohen’s *d*. For sample sizes (>20), the results for both statistics are roughly equivalent. In the present study, we report effect sizes utilizing both methods as our sample size is (> 20). Statistical significance was set at *p* < .05.

## 3 RESULTS

### 3.1 Comparison of Demographic and Clinical Characteristics Between Groups

Results revealed no statistically significant difference among the three groups on the variable age (F_(2,36)_ = 3,044, *p* = .060), gender (*x*^2^ = 1,293, *p* = .075), and years of education (F_(2, 36_ = 1.168, *p*= .323). As for the two clinical groups, a statistically significant difference between the two clinical groups was found on disease duration (t_(25)_ = -.5004, *p* = <.001), with the SPMS group being diagnosed significantly earlier compared to the RRMS group. Statistically significant differences were also found on the variables FSS (t _(25)_= -.9110, *p*<.001), with the SPMS group showing higher fatigue levels compared to the RRMS group. We also found that disability level (EDSS) differed significantly between the two groups [*z*= -2.826, *p*< .001], with the SPMS group showing more severe disability. Severity of depression (t _(25)_= -4.494, *p* = .489) and WASI vocabulary score (t _(25)_= -5.098, *p* = .275) on the contrary were not significantly different between the two groups. (see Table **1**).

### 3.2 Comparison of Neuropsychological and Language Performance Among the Groups

Our results showed that there was a main group effect on the RAVLT (trials 1-5; mean of total words recalled in the five trials) (F_(2,36)_ = 12.606, *p* = .000); RAVLT (trial 1; mean of total words recalled in the first trial) (F_(2,36)_ = 2.804, *p* = .0035); RAVLT (delayed recall; mean of total words retrieved after a delay period) (F_(2,36)_ = 8.243, *p* = .0011); phonological verbal fluency task (F_(2,36_) = 3.951, *p* = .028); semantic verbal fluency (F_(2,36)_ = 3.906, *p* = .029); TMT part A (F_(2,36)_= 5.680, *p* = .007); TMT part B (F_(2,36)_ = 9.697, *p* = .000); SNST (F_(2,36)_ = 6.278, *p* = .001) and SDMT (F_(2,36)_ = 2.604, *p* = .003).A post-hoc test with Bonferroni correction demonstrated statistically significant differences between the two clinical groups in the RAVLT (Trial 1), the SDMT, the SNST and the TMT B. Statistically significant differences between the RRMS group and the CG were found in the RAVLT (Trial 1), the RAVLT (Total trials 1-5), RAVLT (Delayed Recall), SDMT and SNST. Significant differences were noted on all tests between the SPMS and healthy group on all utilized measures (Table **2**).

When comparing effect sizes for the differences noted between the two MS clinical subgroups we found large effect sizes on episodic memory (learning curve), semantic fluency, processing speed and executive function (set-shifting). Comparison between RRMS patients and controls revealed large effect sizes on episodic memory (learning curve and delay recall), attention / response inhibition and executive function (set-shifting). Comparison between SPMS patients and controls showed large effect sizes on all utilized measures. All other effects sizes between groups were either small or medium (Table **3**).

### 3.3 Frequency of Impairment in Various Measures for the MS Clinical Groups

Patients failed a particular measure (test) if they scored 1.5 standard deviation below the performance of the control group. The highest frequencies of impairment for the RRMS group were observed on measures of processing speed (SDMT) (60%), episodic memory (encoding) (46.6%) and executive function (set-shifting) (46.6%). For the SPMS group the highest frequencies of impairment were noted on processing speed (SDMT) (83.3%),episodic memory (encoding) (75%), and executive function (set-shifting) (75%) (Table **4**).

### 3.4 Correlations Between Duration and Severity of the Disease and Neuropsychological-Language Tests

The association between disease severity - duration and performance on the neuropsychological-language tests was explored using the Pearson method as most of our variables were normally distributed. Positive correlations were found between disease severity (EDSS) and the duration of the disease (*r* = 0.745, *p* = .000) and between disease severity and performance on the SDMT (mental processing speed and working memory) (*r* = 0.605, *p*< .001). A moderate correlation was noted between disease severity and performance on part B (set shifting) of the TMT (*r* = 0.472, *p* = .013). As our sample is relatively small, only the EDSS (disease severity) variable did not follow the normal distribution and therefore we repeated the correlation analysis with the non -parametric Spearman’s rho. We found that the significant correlations between *EDSS *and *duration of disease* (Pearson *r* = 0.745,* p* = .000, Spearman’s rho *r *= 0.731. p = .000), *EDSS and TMT B* (Pearson *r* = 0.472,* p* = .013, Spearman’s rho *r *= 0.415, p = .014), remained significant in the same direction and strength of the relationship.

## 4 DISCUSSION

According to the literature, about 40-65% of the patients with MS will, at some point of the disease course, develop cognitive deficits, regardless of the duration and severity of the disease [7]. This cognitive decline, which appears to worsen over time [41], has a significant negative impact on patients' daily functioning, which can even lead to the loss of employment and ultimately to a lower quality of life [42].

An issue that remains ambiguous about cognitive impairment in MS and especially in Greek patients is whether cognitive deficits in the various clinical types of MS differ qualitatively and quantitatively. The present study attempted to fill this gap, by examining and comparing the cognitive and linguistic functions among patients with RRMS, SPMS, and healthy adults. For the evaluation of cognitive - linguistic functions both neuropsychological and language tests were used, which have been translated, adapted and standardized on the Greek population with satisfactory discriminatory validity in patients with MS [7, 26, 33].

Initial comparisons revealed that the three groups did not differ on demographic characteristics, which could affect performance on the tests. Age, gender and years of education were well matched between the three groups. In contrast to the above parameters, statistically significant differences between the two clinical groups were found on disease duration, level of disability and level of fatigue, with the SPMS group being diagnosed significantly earlier than the RRMS group, and, at the same time, having a more severe disability and higher levels of fatigue. Although these results were anticipated, due to the progressive nature of the SPMS group, to statistically compensate for the contribution of these three factors on performance in the cognitive - linguistic tests, we used ANCOVAs in the final comparison of performance in the various tests. In contrast, the severity of depression and premorbid intelligence level were well matched between the two clinical groups.

Comparison of the various tests revealed differences between the RRMS and SPMS group on the verbal encoding of new items, with a much poorer performance of the latter group. However, overall, the two clinical groups did not differ on the consolidation and delayed recall of the items. Moreover, the SPMS group required more time and had more difficulty in processing the new material satisfactorily. Furthermore, large effect sizes on the differences between the two clinical groups were noted on processing speed and verbal encoding, thereby strengthening the size of the revealed difference between RRMS and SPMS patients in the respective cognitive domains.These findings are consistent with those of Chiaravalloti and DeLuca [44] who reported that initial encoding deficits are very common in MS patients, due to difficulty in initial processing and low mental processing speed. These deficits appear to be aggravated in the progressive form of the disease and especially the SPMS type. As for executive functions, the patients with SPMS had statistically significant lower performance in the tests that needed switching and inhibition skills. The results of the present study are in line with the results of the recent, Greek study conducted by Katsari *et al*. [12], according to which only patients with SPMS were found to have deficits in episodic memory and executive functions. Thus, the hypothesis that the progressive type of disease shows a relatively greater reduction in cognitive function, compared to the RRMS type, was confirmed.

Significant differences were revealed between the RRMS and the healthy group on initial verbal word encoding, word consolidation and delayed recall, confirming that MS patients, regardless of the clinical subtype, have deficient verbal episodic memory [45]. Furthermore, significant differences were found between the healthy group and SPMS patients on all cognitive domains that were assessed.This finding is in keeping with the Papathanasiou *et al*. [51], study that reported similar results between their SPMS and healthy participants. We also noted high frequencies of impairment on individual measures of processing speed, verbal encoding and set-shifting in both clinical groups, although, SPMS patients recorded higher impairment percentages on all utilized measures.

On the contrary, no significant differences were attested between the RRMS and healthy group in the tasks that examined verbal fluency. This finding suggests that verbal fluency functions are mainly affected in the late stages of the disease, especially in the progressive types. Contrary to the results of our study, Henry and Beatty [18] reported that patients with RRMS presented a substantially lower performance on verbal fluency compared to controls, while patients with SPMS showed more severe deficits compared to patients with RRMS. The explanation for the different findings between the two studies might lie in the strategies utilized for maximizing word production. It appears that performance in these tests depends not only on purely linguistics factors, but is also influenced by participants' abilities to access their mental lexicon. Therefore, it could be that the RRMS and SPMS patients who took part in our study did not differ significantly in their ability to retrieve the stored words and their properties and thus used this ability to compensate for a potential difficulty in verbal output.As for the comparison between the SPMS group and controls, differences were found on all measures, indicating possibly more severe neurodegeneration in this clinical form of the disease, leading to more severe deficits in a wider range of cognitive functions [47].

The findings of our study comply with the typical profile of cognitive deterioration expected in MS patients *i.e*. deficits in mental processing ability and processing speed, episodic memory and executive functions, with relative preservation of language skills [43, 46].Moreover, our results revealed that a higher percentage and more severe levels of cognitive decline tend to occur in the progressive phase of the disease *i.e*. SPMS patients in this case.

Regarding the second hypothesis, we found a relatively large correlation between disease duration and disability severity, disease severity and mental processing speed and a moderate association between disease severity and episodic memory and executive function deficits. The high correlation found between the duration and the severity of the disease was expected since the degree of disability increases progressively over the years in this disease [48]. The high correlation attested between cognitive processing speed/working memory and the severity of the disease, is an exception in the literature, as far as the association between disability and cognitive functions is concerned. According to Lezak *et al*. [17], however, only these functions appear to be adequately related to the degree of disability. The moderate association that was established between the executive function of set-shifting and the disability severity of the disorder,provides support to our second hypothesis, according to which mild to moderate correlations were expected to be found between disease duration, severity and performance on the neuropsychological- language battery ([41]). No other significant associations were noted between the above variables. This could be because in our sample several patients, especially in the SPMS group, might have lesions mainly in the spinal cord, which causes an increased degree of motor disability but no cognitive impairments [49].

Although the study has several strengths, including the well-matched baseline demographic and clinical characteristics; the validated for Greek MS patients’ comprehensive neuropsychological – language battery that was utilized;the strict exclusion and inclusion criteria;and the absence of comorbid conditions that may have biased the study outcome measures, it does have some potential limitations. Firstly, the sample size of the three groups is relatively small, partly influencing the various findings. Secondly, the study sample does not represent the whole MS population and subtypes (*i.e*. clinically isolated syndromes and primary progressive MS patients were not included), thereby limiting generalization of the findings. A final limitation of the study is that language testing was limited to verbal fluency, and we did not consider other language domains such as syntax and discourse, which involve more complex linguistic processes and may have differed between the two clinical groups.

## CONCLUSION

In the present study, the performance of two clinical groups with MS (RRMS and SPMS) and a group of healthy adults were assessed on both cognitive and language tasks. The results revealed that patients with SPMS demonstrated poorer performance on certain cognitive tasks (initial word encoding, mental processing speed and executive functions) and not worse overall global cognitive impairment, compared to patients with RRMS. Furthermore, the RRMS group had lower performance compared with the controls in the initial encoding of verbal material, consolidation of words and delayed recall. The most significant cognitive differences, however, were attested between the SPMS patients and the healthy participants.This finding confirms the assumption that MS patients, irrespective of the clinical subtype, have more severe deficits than healthy participants, which become increasingly worse as the severity of disease proceeds and RRMS patients convert to SPMS.On the contrary,the pattern of cognitive impairment remains relatively stable. Regarding language performance, only the SPMS patients differed from the healthy participants on measures of verbal fluency. In this respect, in order to investigate the impact of progressive MS in comparison to relapsing remitting MS on linguistic function, we recommend that future studies utilize measures that require higher language processing (*e.g*., understanding metaphors, reconstructing sentences, and making inferences).

## Figures and Tables

**Table 1:** Demographic and clinical characteristics of the three groups mean: (standard deviation).

–	RRMS n=15	SPMS n=12	CG n=12
Gender (Women)	12 (80%)	9 (75%)	9 (75%)
Age (in Years)	43.60 (9.74)	48.67 (8.07)	41.00 (3.34)
Years of Education	11.67 (2.61)	11.75 (3.36)	13.08 (1.50)
Disease Duration	9.20 (3.70)	18.42 (5.82)	–
WASI Vocabulary Scale T-score	41.50	42.10	–
EDSS Median (Range)	3.5 (1.5 – 4.0)	6.25 (6.0 -7.5)	–
FSS	4.02 (1.35)	4.80 (1.80)	–
BDI-FS	3.67 (2.45)	3.80 (2.46)	–

RRMS= Relapsing RemittingMultiple Sclerosis; SPMS = Secondary Progressive Multiple Sclerosis; CG= Healthy control group; EDSS = Expanded Disability Status Scale; FSS = Fatigue Severity Scale; BDI-FS = Beck Depression Inventory –Fast Screen; WASI = Wechsler abbreviated Scale of Intelligence Note: Intelligence level was estimated by administering the vocabulary and matrix reasoning subscales of the Wechsler abbreviated scale of intelligence (WASI), Greek adapted version (Messinis *et al*., 2009). The vocabulary subscale is a good measure of crystallized intelligence, correlates well with general intellectual ability and is relatively insensitive to cortical insults (*i.e*., a good measure of premorbid intellectual ability). The matrix reasoning subscale is a measure of nonverbal fluid reasoning and correlates well with general intellectual ability. These two subscales yield an estimated full-scale IQ All variables investigated for normality with the Shapiro-Wilk test, were normally distributed, *p* < 0.05, except EDSS which was not normally distributed, *p* .> 0.05

**Table 2:** Comparison of neuropsychological and language performance between the three groups: means (standard deviation).

–	–	–	–	* p value *		
–	RRMS (n=15)	SPMS (n=12)	CG (n=12)	RRMS vs. SPMS	RRMS vs. CG	SPMS vs. CG
RAVLT Trial 1	6.90 (1.45)	6.05 (1.20)	7.50 (1.90)	.015*	.030*	.025*
RAVLT Trials 1 to 5	47.87 (6.41)	41.33 (7.57)	55.08 (6.14)	.050	.026*	.000*
RAVLT Delayed Recall	10.10 (2.15)	9.65 (1.35)	12.30 (2.60)	.067	.013*	.001*
Phonological Fluency	27. 25 (12.51)	22.75 (12.18)	35.42 (7.96)	.915	.206	.027*
Semantic Fluency	44.13 (10.41)	37.08 (5.33)	48.00 (11.94)	.208	.934	.028*
SDMT	45. 90 (7.50)	37.25 (6.50)	52. 15 (9.65)	.000*	.003*	.037*
SNST	65.70 (9.50)	60. 20 (7.40)	105.15 (2.85)	.030*	.000*	.001*
TMT - A	52.13 (17.38)	69.06 (18.07)	44.33 (15.46)	.070	.846	.007*
TMT - B	97.72 (30.12)	69.06 (17.05)	44.33 (15.45)	.003*	1.00	.001*

RAVLT Trial 1= Rey Auditory Verbal Learning Test Trial 1; RAVLT Trials 1-5 = Rey Auditory Verbal Learning Test Trial 1-5; RAVLT Delayed Recall= Rey Auditory Verbal Learning Test Delayed Recall; SDMT = Symbol Digits Modalities Test; SNST = Stroop Neuropsychological Screening Test; TMT A = Trail Making Test part A; TMT A = Trail Making Test part B Significant difference among groups on that variable (*p* <.05)*, all other comparisons were not significantly different.

**Table 3:** Effect sizes for differences between MS subgroups (RRMS, SPMS) and controls.

–	RRMS versus SPMS Cohen’s *d * Hedges’ *g *		RRMS versus Controls Cohen’s *d* Hedges’ *g*		SPMS versus Controls Cohen’s *d* Hedges’ *g*	
RAVLT Trial 1	0.638	0.631	0.355	0.360	0.912	0.936
RAVLT Trials 1 to 5	0.932	0.941	1.148	1.145	1.995	1.970
RAVLT Delayed Recall	0.250	0.244	0.922	0.932	1.279	1.325
Phonological Fluency	0.364	0.363	0.779	0.760	1.231	1.202
Semantic Fluency	0.852	0.824	0.345	0.348	1.181	1.231
SDMT	1.232	1.222	0.723	0.734	1.811	1.853
SNST	0.645	0.636	5.625	5.362	8.016	7.681
TMT - A	0.954	0.957	0.474	0.470	1.470	1.457
TMT - B	1.171	1.136	2.230	2.156	1.520	1.511

RAVLT Trial 1= Rey Auditory Verbal Learning Test Trial 1; RAVLT Trials 1-5 = Rey Auditory Verbal Learning Test Trial 1-5; RAVLT Delayed Recall= Rey Auditory Verbal Learning Test Delayed Recall; SDMT = Symbol Digits Modalities Test; SNST = Stroop Neuropsychological Screening Test; TMT A = Trail Making Test Part A; TMT A = Trail Making Test Part B; Note: For Cohen’s *d* and Hedges’ *g*, an effect size of 0.2 to 0.3 is considered a “small” effect, 0.5 a “medium effect” and ≥ 0.8 a “large effect”.

**Table 4:** Frequency of impairment in respective measures for RRMS and SPMS patients: number and percent (%).

–	RRMS ( n=15)		SPMS (n=12)	
RAVLT Trial 1	7	(46.6%)	9	(75.0%)
RAVLT Trials 1 to 5	6	(40.0%)	8	(66.6%)
RAVLT Delayed Recall	4	(26.6%)	7	(58.3%)
Phonological Fluency	4	(26.6%)	7	(58.3%)
Semantic Fluency	3	(20.0%)	6	(50.0%)
SDMT	9	(60.0%)	10	(83.3%)
SNST	4	(26.67%)	6	(50.0%)
TMT - A	5	(53.3%)	7	(53.3%)
TMT - B	7	(46.6%)	9	(75.0%)

RAVLT Trial 1= Rey Auditory Verbal Learning Test Trial 1; RAVLT Trials 1-5 = Rey Auditory Verbal Learning Test Trial 1-5; RAVLT Delayed Recall= Rey Auditory Verbal Learning Test Delayed Recall; SDMT = Symbol Digits Modalities Test; SNST = Stroop Neuropsychological Screening Test; TMT A = Trail Making Test Part A; TMT A = Trail Making Test Part B *Note:* Patients failed a measure (test) if they scored *1.5 standard deviation* below the performance of the control group.
